# Carboxymethyl Chitosan-Functionalized Polyaniline/Polyacrylonitrile Nano-Fibers for Neural Differentiation of Mesenchymal Stem Cells

**DOI:** 10.1007/s12010-023-04526-6

**Published:** 2023-04-18

**Authors:** Sahar Arbab Solimani, Shiva Irani, Marjan Mohamadali, Hadi Bakhshi

**Affiliations:** 1 Department of Biology, Science and Research Branch, Islamic Azad University, Tehran, Iran; 2 Department of Life Science and Bioprocesses, Fraunhofer Institute for Applied Polymer Research IAP, Geiselbergstraße 68, 14476 Potsdam-Golm, Germany

**Keywords:** Carboxymethyl chitosan, Polyaniline, Polyacrylonitrile, Surface-functionalization, Neural differentiation

## Abstract

Electroconductive scaffolds based on polyaniline (PANi)/polyacrylonitrile (PAN) were fabricated and surface-functionalized by carboxymethyl chitosan (CMC) as efficient scaffolds for nerve tissue regeneration. The results of scanning electron microscopy (SEM), Fourier-transform infrared (FTIR) spectroscopy, and water contact angle measurement approved the successful fabrication of CMC-functionalized PANi/PAN-based scaffolds. Human adipose-derived mesenchymal stem cells (hADMSCs) were cultured on the scaffolds for 10 d in the presence or absence of β-carotene (βC, 20 µM) as a natural neural differentiation agent. The MTT and SEM results confirmed the attachment and proliferation of hADMSCs on the scaffolds. The expression of MAP2 at the mRNA and protein levels showed the synergic neurogenic induction effect of CMC-functionalization and βC for hADMSCs on the scaffolds. The CMC-functionalized nanofibrous PANi/PAN-based scaffolds are potential candidates for nerve tissue engineering.

## Introduction

Electroconductive and biocompatibility polymers such as polyaniline (PANi), polypyrrole, polythiophene, and their derivatives have found wide applications in biomedical fields including bioactuators, biosensors, neural implants, drug delivery systems, and tissue engineering [1]. Tissue engineering uses the proper scaffolds to help regenerate/repair the missing/damaged tissues. Biocompatibility, biodegradability, three-dimensional (3D) structure, and interconnected porosity of scaffolds are important parameters to support cell attachment, proliferation, and differentiation [2, 3]. The electroconductivity of scaffolds is important in tissue engineering since it improves the propagation of bioelectrical signals to stimulate cell attachment and tissue formation on them [1]. Electroconductive polymers are used as biomaterials to stimulate muscle, bone, cardiac, and nerve tissues, although their in vivo applications may be constrained by their non-degradability [4–6].

PANi is one of the most promising electroconductive polymers having a wide range of applications in bioengineering and biomedicine. PANi appears to be simple to synthesize and display a variety of electroconductivities, biocompatibility, good environmental stability, and a distinctive and simple doping method [7, 8]. Despite all these positive qualities, PANi has low mechanical strength hindering its applications, especially as tissue engineering scaffolds [7]. The mechanical stability of PANi scaffolds can be improved via the combination of another polymer with high mechanical strength such as polyacrylonitrile (PAN) [8–11]. PAN is a cost-effective and thermally and chemically stable engineering polymer generally used for textile purposes. Using the electrospinning process, the PANi/PAN nanofibrous scaffolds provided adequate support for cell adhesion, proliferation, and differentiation into muscle-like cells [8–10].

Electrospinning is a promising technique to fabricate nanofibrous scaffolds from a wide range of natural/synthesis polymers including electroconductive polymers [12]. The high porosity and high surface-to-volume ratio, as well as, the appropriate mechanical stability of electrospun nanofibers make them attractive scaffolds for neural tissue engineering. The nanofibrous nature of electrospun scaffolds mimics the structure and composition of collagen fibers within native ECM, which facilitates the migration, attachment, and proliferation of cells as well as the transition of nutrients and biochemical factors [13]. Meanwhile, the composition, morphology, structure, and 3D architecture of electrospun scaffolds can be easily controlled by choosing adequate material and processing parameters to regulate their biological functions and specific light/electric/magnetic properties [14]. Nanofibers with various dimeters (40-2000 nm) can be fabricated by choosing a suitable combination of polymers and solvents [13].

Chitosan has been widely utilized in medication delivery, tissue engineering, and biomedical implants due to its exceptional benefits like biocompatibility, biodegradability, antifungal, antibacterial, and anticancer properties [15, 16]. However, it has shortcomings such as poor mechanical stability and water-insolubility [15, 16]. Chitosan can be converted into water-soluble forms such as carboxymethyl chitosan (CMC) [17–20]. The carboxymethylation of amine and hydroxyl groups of chitosan improves the water-solubility and processability for tissue engineering applications [21–24]. Sharifi et al. [23] have reported that the incorporation of CMC into polycaprolactone (PCL)-based nanofibrous scaffolds improved their surface hydrophilicity/wettability and promoted the attachment of human osteoblast cells (MG63) and consequently their proliferation.

Beta-Carotene (βC) is a plentiful provitamin A carotenoid found in dark green, yellow, and orange fruits and vegetables [20, 25–27]. Because of conjugated double bonds, βC has electrical activity [28, 29]. The ability of βC to induce neurogenesis for stem cells has been reported recently [30–34]. This research aimed to fabricate electroconductive nanofibers based on PANi/PAN via the electrospinning process and then surface-functionalization with CMC. βC was used as a natural bioactive component to induce neural differentiation for stem cells. Here, we investigated how a combination of CMC-functionalized PANi/PAN nanofibers and βC might imitate the structure of extracellular matrix (ECM) and facilitate cell proliferation and neural differentiation of human adipose-derived mesenchymal stem cells (hADMSCs).

## Experimental

### Materials

Polyaniline (PANi, Emeraldine base, M_W_ = 100,000 Da), polyacrylonitrile (PAN, M_W_ = 100,000 Da), camphor sulfonic acid (CSA), and *N*,*N*-dimethylformamide (DMF), acetic acid, sodium hydroxide, isopropanol, monochloroacetic acid, ethanol, 3-[4,5-dimethylthiazol-2-yl]-2,5-diphenyltetrazolium bromide (MTT), dimethyl sulfoxide (DMSO), glutaraldehyde and 4’,6-diamidino-2-phenylindole (DAPI) were purchased from Sigma-Aldrich. Chitosan (CTS) extracted from the crab shell with a medium weight and degree of deacetylation of > 90% was bought from Bio Basic Inc. (Canada). Dulbecco’s modified eagle’s medium (DMEM), fetal bovine serum (FBS), phosphate-buffered saline (PBS), and trypsin/EDTA solution were purchased from Gibco (USA).

### Synthesis of CMC

Carboxymethyl chitosan (CMC) was synthesized according to our previous reports [21–23]. Briefly, CTS (1 g) was purified by dissolving in acetic acid solution (1%, 40 mL) at room temperature, precipitating with sodium hydroxide solution (1 M, 50 mL), and washing with deionized water and later isopropanol. The purified chitosan was added to sodium hydroxide solution in isopropanol (0.1 g/mL, 20 mL) and mechanically stirred for 5 h to completely be dissolved. Afterward, a monochloroacetic acid solution in isopropanol (0.4 g/mL, 5 mL) was added dropwise to the chitosan solution and stirred at room temperature for 8 h. Finally, the resulting precipitate was filtered, washed with a mixture of ethanol/deionized water (1/3, v/v) three times, and dried in a vacuum oven at room temperature.

### Fabrication of Nanofibers

PANi (0.01 g) and CSA (0.01 g) were added to DMF (3 mL), while PAN (0.39 g) was separately added to DMF (2 mL) and stirred at room temperature overnight for complete desolvation. The PANi/CSA solution was filtered, mixed with the PAN solution, and stirred at room temperature for 2 h. The electrospinning process was performed on a Nano Spinner apparatus (Iran) operating at a voltage of + 15 kV, a flow rate range of 0.2 mL/h, and a nozzle-to-collector distance of 16 cm. The PANi/PAN nanofibers were collected on a drum covered with aluminum foil. The electrospun mats were cut in 0.5 × 0.5 cm^2^ dimensions before use.

### CMC-Functionalization of Nanofibers

The PANi/PAN mats (0.5 × 0.5 cm^2^) were then immersed in a solution of ethanol/deionized water (1/1, v/v) for 3 h and rinsed with a large volume of deionized water to eliminate any contamination. The washed mats dried under the fume hood. CMC was added to deionized water at three concentrations (1%, 2%, and 3% w/v) and stirred for 24 h to completely be dissolved. The PANi/PAN mats were placed in CMC solutions for 1 h, immersed in glutaraldehyde solution (0.5%, w/v) at 37 °C for 2 h, and rinsed with a large volume of deionized water to remove the uncrosslinked CMC. The surface-functionalized mats (PANi/PAN/CMC1%, PANi/PAN/CMC2%, and PANi/PAN/CMC3%, respectively) were dried in a vacuum oven at 40 °C for 24 h.

### Characterization of Scaffolds

The morphology and fiber diameter of mats were studied by scanning electron microscopy (SEM, Tescan, Mira3, Czech Republic). All samples were coated with a gold layer before microscopy. The diameter of fibers was measured using Image J software (version 1.41). Fourier-transform infrared (FTIR) spectroscopy (Equinox 55, Bruker, Germany) was employed to characterize the chemical structure of mats.

### Cell Seeding on Scaffolds

Human adipose-derived mesenchymal stem cells (hADMSCs) obtained from the Iranian Biological Resource Center (Tehran, Iran) were cultured in DMEM medium supplemented with FBS (10%) in a humidified incubator at 37 °C under CO_2_ (5%). Each side of the scaffolds was sterilized under UV radiation for 20 min. The hADMSC suspension (100 µL, 10^4^ or 10^6^ cells) was placed on the scaffold (0.5 × 0.5 cm^2^) in a 96-well tissue culture plate in triplicate and incubated at 37 °C under CO_2_ (5%) for 2 h for the attachment of cells. Finally, the seeded scaffolds were cultured in nutrient DMEM (1 mL) with or without βC (20 µM) in an incubator at 37 °C under CO_2_ (5%). The media were replaced every three days. A tissue culture plate (TCP) without any scaffold was used as a control.

### Biocompatibility Assays

The morphological changes of the seeded cells (10^4^ cells/0.5 × 0.5 cm^2^ scaffold) up to 72 h of incubation were observed under an optical microscope (Olympus, Japan). The proliferation of the seeded cells (10^4^ cells/0.5 × 0.5 cm2 scaffold) up to 10 d of incubation was assessed through MTT assay. For this purpose, the medium of each sample (n = 3) was replaced with 200 µL of MTT solution (5 g/L) followed by incubation at 37 °C for 3 h. The formed formazan crystals were dissolved in DMSO and the optical density (OD) of the solution was measured at 570 nm on an Eliza reader instrument (Bio-Tek ELx 800).

The morphology and attachment of the cells (10^4^ cells/0.5 × 0.5 cm^2^ scaffold) cultured for 72 h were studied by SEM. To this end, the cells were washed with PBS and fixed in a glutaraldehyde solution (2.5%) for 2 h, dehydrated in a series of ethanol solutions (60%, 70%, 80%, 90%, and 100%), and dried at room temperature.

### Neural Differentiation Assays

The expressions of the *Microtubule-associated protein 2* (*MAP2*) gene as a specific neural marker at the mRNA level in the cells (10^6^ cells/0.5 × 0.5 cm2 scaffold) cultured for 10 d were evaluated by quantitative reverse transcription-polymerase chain reaction (qRT-PCR). After washing twice with PBS, the total RNA of the cells was extracted by a total RNA isolation kit (Ribo-Ex, GeneAll, South Korea) and converted to cDNA using an easy cDNA synthesis kit (Parstous Biotechnology, Iran) according to the manufacturer’s protocols. Finally, the RT-PCR process was performed on a thermal cycler (Rotor-Gene Q, Qiagen, USA) using a master mix reagent (2X PCR Master Mix, Biofact Co., South Korea). The expression of the *Glyceraldehyde 3-phosphate dehydrogenase* (*GAPDH*) gene was evaluated as a reference gene. The expressions of the *MAP2* gene were quantified by normalizing them with the expressions of the *GAPDH* gene. The primers designed for RT-PCR were as follows; *MAP2*; F: 5’-TAAGGATCAAGGCGGAGCAG-3’, R: 5’-AGACACCTCCTCTGCTGTTTC-3’, *GAPDH*: F: 5’-CTCATTTCCTGGTATGACAACG-3’, R:5’-CTTCCTCTTGTGCTCTTGCT-3’.

The expressions of MAP2 protein in the cells (10^4^ cells/0.5 × 0.5 cm^2^ scaffold) cultured for 10 d were assessed by immunocytochemistry (ICC) assay. The scaffolds were washed with PBS, fixed with paraformaldehyde solution (4%) at 4 °C for 20 min and then at room temperature for 5 min, washed with PBS, permeabilized with Triton X-100 (4%) for 10 min, and washed again with PBS. The permeabilized cells were immersed in FBS for 45 min to block the nonspecific binding sites, incubated with the primary antibody for the *MAP2* protein (Santa Cruz Biotechnology, USA) at 4 °C overnight, washed with PBS, incubated with goat serum (5%) for 45 min, and incubated with secondary antibody conjugated with FITC (Abcam, USA) for 30 min, and washed again with PBS. Eventually, the nuclei of cells were stained with DAPI, washed twice with PBS, and observed under a fluorescent microscope (Olympus LS, IX53, Japan).

### Statistical Analysis

The results expressed as mean ± SD are representing at least three independent experiments. The differences between groups were analyzed using the one-way ANOVA method after testing for homogeneity of variances by the PASW Statistics program package (version 19, SPSS Inc., USA). The statistical significance was assigned as * for *p* ≤ 0.05, ** for *p* ≤ 0.01, and *** for *p* ≤ 0.001.

## Result and Discussion

### Fabrication of Scaffolds

PANi presents in three different redox forms including fully-reduced-state Leucoemeridine, half-oxidized-state Emeraldine, and fully-oxidized-state Pernigraniline. Emeraldine is the most applicable form of PANi due to its high stability at room temperature and high electroconductivity in the protonated form (Emeraldine salt) [8]. Here Emeraldine base was protonated/doped with CSA, which makes it also soluble in common organic solvents such as DMF. Meanwhile, a high level of room-temperature electroconductivity (300 ± 30 S/cm) can be obtained [35].

**Fig. 1:**
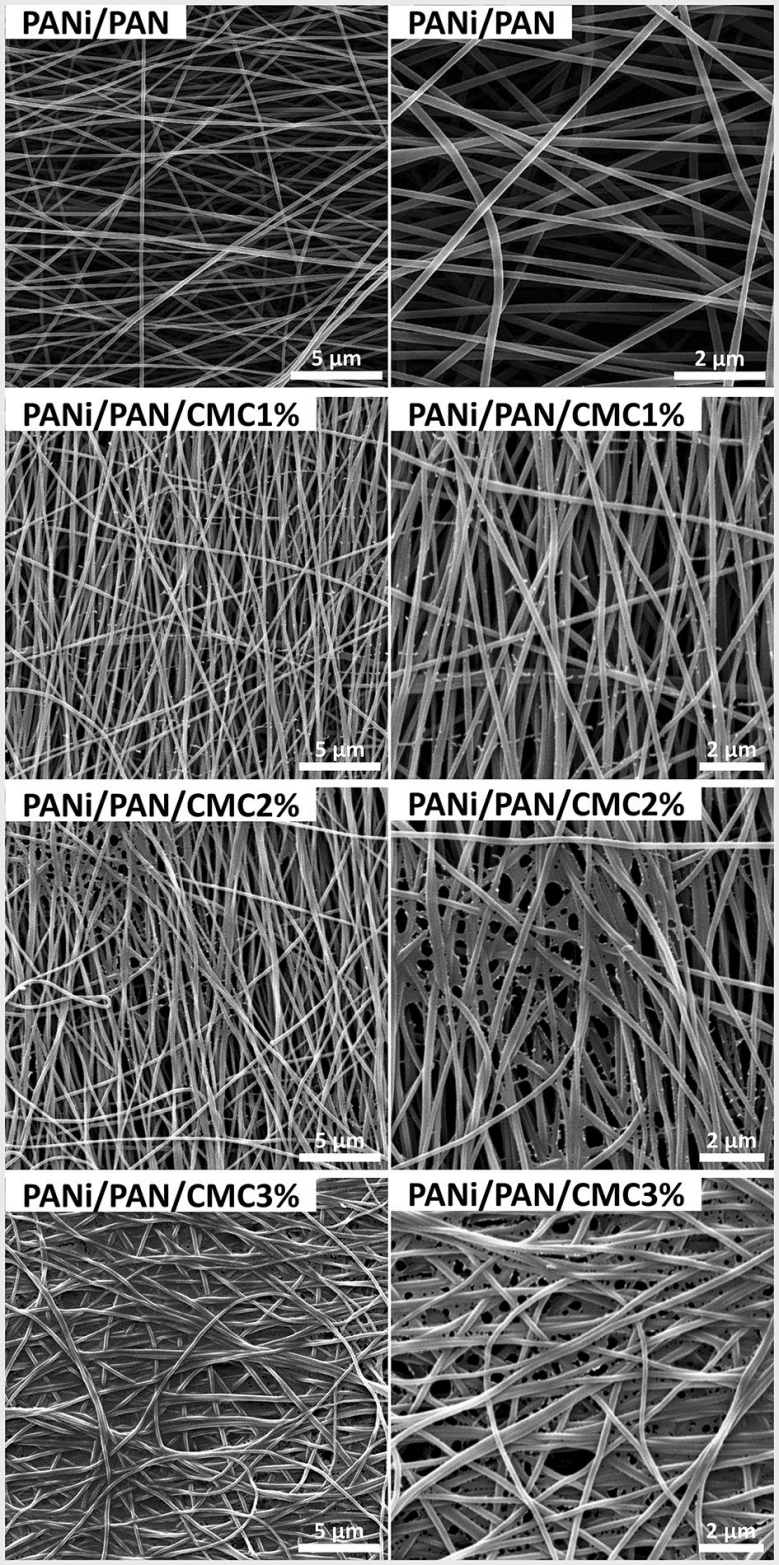
SEM images of virgin and CMC-functionalized PANi/PAN nanofibers

Here, PANi/PAN (2.6/100, wt.wt.) fibrous mats were fabricated via the electrospinning process. In PANi/PAN nanofibers, the hydrogen bonding between the amine groups of PANi and the nitrile groups of PAN is possible [11]. SEM images (Fig. 1) showed that the electrospun PANi/PAN mats are bead-free and random-oriented nanofibers with an average diameter of 183 ± 37 nm, which presented a highly micro-porous structure with large interconnected cavities suitable for providing the appropriate biological conditions for the migration, attachment, and proliferation of cells. A similar nanofibrous PANi/PAN (2.1/100, wt./wt.) scaffold developed by Hosseinzadeh et al. [8] showed an elastic modulus of 146.19 ± 7.22 MPa, a maximum stress of 1.69 ± 0.11 MPa, and a strain at break of 56.3 ± 3.4%.

The surface of PANi/PAN nanofibers was functionalized with CMC. For this purpose, the water-soluble CMC was coated on the surface of nanofibers and crosslinked with glutaraldehyde. The crosslinking occurred by reacting the amine groups of CMC and the aldehyde groups of glutaraldehyde [17, 21]. The surface of PANi/PAN nanofibers was coated using CMC solutions with three various concentrations (1%, 2%, and 3% w/v) resulting in scaffolds PANi/PAN/CMC1%, PANi/PAN/CMC2%, and PANi/PAN/CMC3%, respectively, to examine the effect of surface-immobilized CMC contents on the attachment, proliferation, and differentiation of cells.

**Fig. 2:**
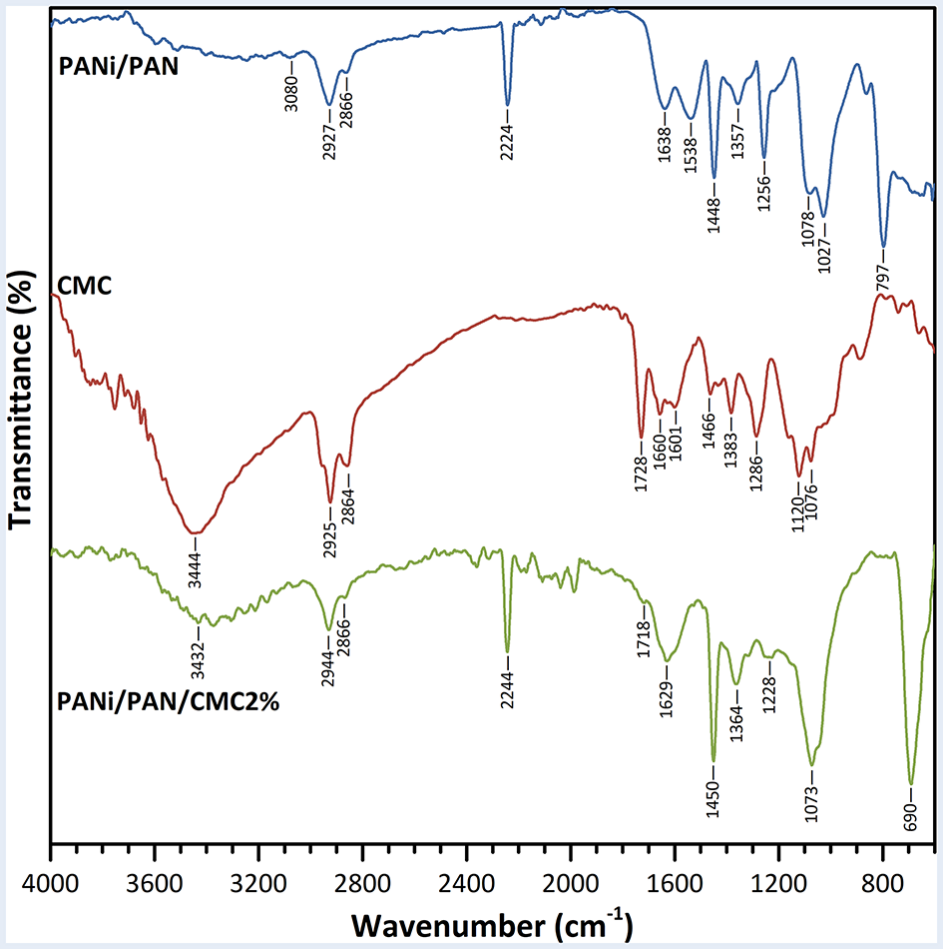
ATR-FTIR spectra of PANi/PAN nanofibers, CMC, and PANi/PAN/CMC2% nanofibers

FTIR spectroscopy was used to study the CMC immobilization on the surface of PANi/PAN nanofibers (Fig. 2). The FTIR spectrum of PANi/PAN nanofibers showed the characteristic peaks of PAN at 2927 and 2866 cm^− 1^ (C–H, ν), 2224 cm^− 1^ (C ≡ N, ν), and 1448 cm^− 1^ (CH_2_, δ) [8, 9, 36]. In addition, a week and wide peak at 3200–3400 cm^− 1^ (N–H, ν, free imine groups), a week peak at 3080 cm^− 1^ (= C–H, ν), and two peaks at 1538 cm^− 1^ (N–H, δ) and 1256 cm^− 1^ (C–N, ν) were detected attributing to PANi [9, 36]. The FTIR spectrum of CMC showed peaks at 3444 cm^− 1^ (O–H, ν and N–H, ν), 2925 and 2864 cm^− 1^ (C–H, ν), 1728 and 1660 cm^− 1^ (C = O, ν, carboxylic acid and carboxylate groups) 1466 and 1383 cm^− 1^ (CH_2_, δ and CH_3_, δ), 1286 cm^− 1^ (C–N, ν), and 1120 and 1076 cm^− 1^ (C–O, ν) [23]. For the PANi/PAN/CMC2% nanofibers, the appearance of characteristic peaks of CMC at 3432 cm^− 1^ (O–H, ν and N–H, ν), 1718 cm^− 1^ (C = O, ν, carboxylic acid) and 1073 cm^− 1^ (C–O, ν) confirmed the successful surface-functionalization process.

SEM images of CMC-functionalized PANi/PAN nanofibers (Fig. 1d) displayed the immobilization of CMC on and between the fibers. The content of surface-immobilized CMC was improved by employing CMC solutions with higher concentrations. The water contact angle of PANi/PAN nanofibers decreased from 49 °C to 45 °C for PANi/PAN/CMC2% nanofibers.

### Biocompatibility

**Fig. 3:**
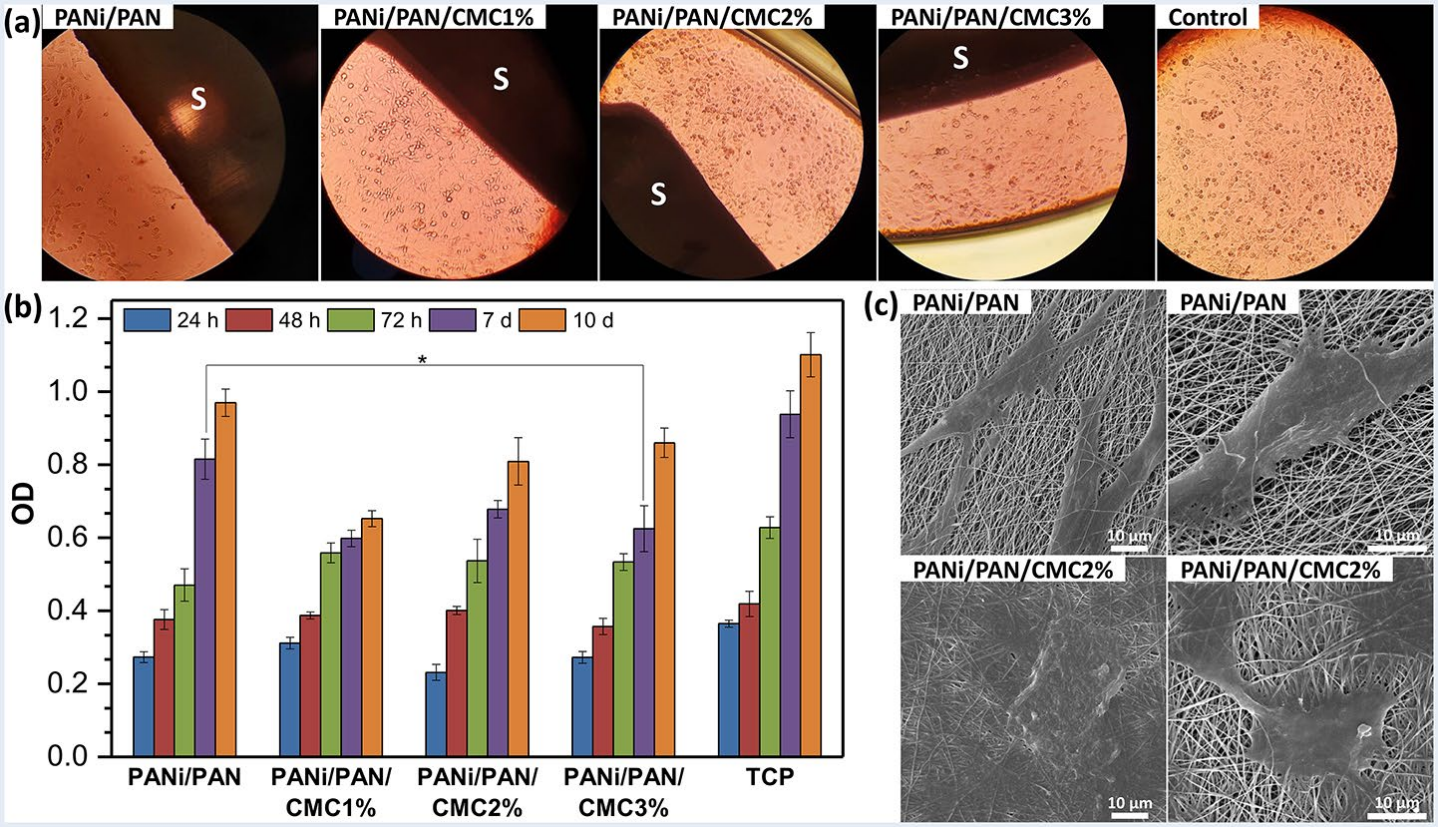
(**a**) Optical microscopy images of hADMSCs (10^4^ cells/0.5 × 0.5 cm^2^ scaffold) cultured on scaffolds for 72 h without any growth factor. (**b**) The cell viability of hADMSCs cultured up to 10 d obtained from MTT assay (*: *p* < 0.05). (**c**) SEM images of hADMSCs cultured for 72 h

The biocompatibility of tissue engineering scaffolds is essential for the attachment of cells to them and later proliferation. Thus, primarily, hADMSCs were cultured on scaffolds PANi/PAN/CMC1%, PANi/PAN/CMC2%, and PANi/PAN/CMC3% for 10 d in the absence of βC. TPC and scaffold PANi/PAN were used as negative controls to see how the presence of a nanofibrous electro-conductive PANi/PAN substrate and surface-immobilized CMC moieties can affect the proliferation of hADMSCs. The daily observation under the optical microscope (Fig. 3a) showed that the hADMSCs on the scaffolds cultured in a nutrient medium without any growth factor were viable with rapid growth during the first 72 h of incubation without any growth factor. The spindle-shaped hADMSCs were pointed in the direction of scaffolds and demonstrated strong cell-cell adhesion. Furthermore, the cell growth on the scaffolds was similar to that on TCP as a control.

The MTT assay was carried out to assess the viability of the seeded cells (Fig. 3b). The MTT results showed that the cells cultured on all scaffolds were viable and proliferated continuously up to 10 d of incubation. Compared to TCP as a control, both CMC-functionalized and virgin PANi/PAN-based scaffolds were able to support cell proliferation up to 10 d and no cytotoxic effect was observed (*p* > 0.5). The biocompatibility of PANi-based scaffolds has been reported earlier [8, 10, 37–39]. Increasing the surface-immobilized CMC contents did not result in any significant change in the proliferation of hADMSCs up to 7 days incubation. However, after 10 days, the proliferation of hADMSCs displayed a gradual increase with rising surface-immobilized CMC content.

SEM images (Fig. 3c) displayed a good spread and attachment of hADMSCs on all scaffolds after 72 h of incubation, in which cells have become more elongated due to the well-established cell-cell and cell-scaffold interactions with actin filaments (invadopodia and filopodia). The surface-functionalization of PANi/PAN nanofibers with CMC resulted in higher cell coverage on the scaffolds demonstrating boosted cell proliferation on them, which is in agreement with OD values obtained from the MTT assay (Fig. 3b). These results confirmed that the fabricated scaffolds are biocompatible and can support the attachment, proliferation, and later differentiation of hADMSCs and mimic the ECM environment for nerve tissue engineering applications.

### Neural Differentiation

Stem cells can differentiate into a variety of cell types. Growth factors like cytokines and hormones are always required for the differentiation of stem cells into specialized cells. βC is a precursor for retinol that can be metabolized to retinoic acid and act in retinoic acid signaling pathways by transmitting the cell-cell signals during the progression of evolutionary processes [40]. Retinoic acid enters the cell nucleus and binds to the target gene via the nucleus receptors [41]. Therefore, βC increases neural differentiation by improving the phosphorylation of extracellular signal-regulated kinases (ERK) [34]. The ability of βC to induce neurogenesis for stem cells has been reported recently [30–34].

**Fig. 4:**
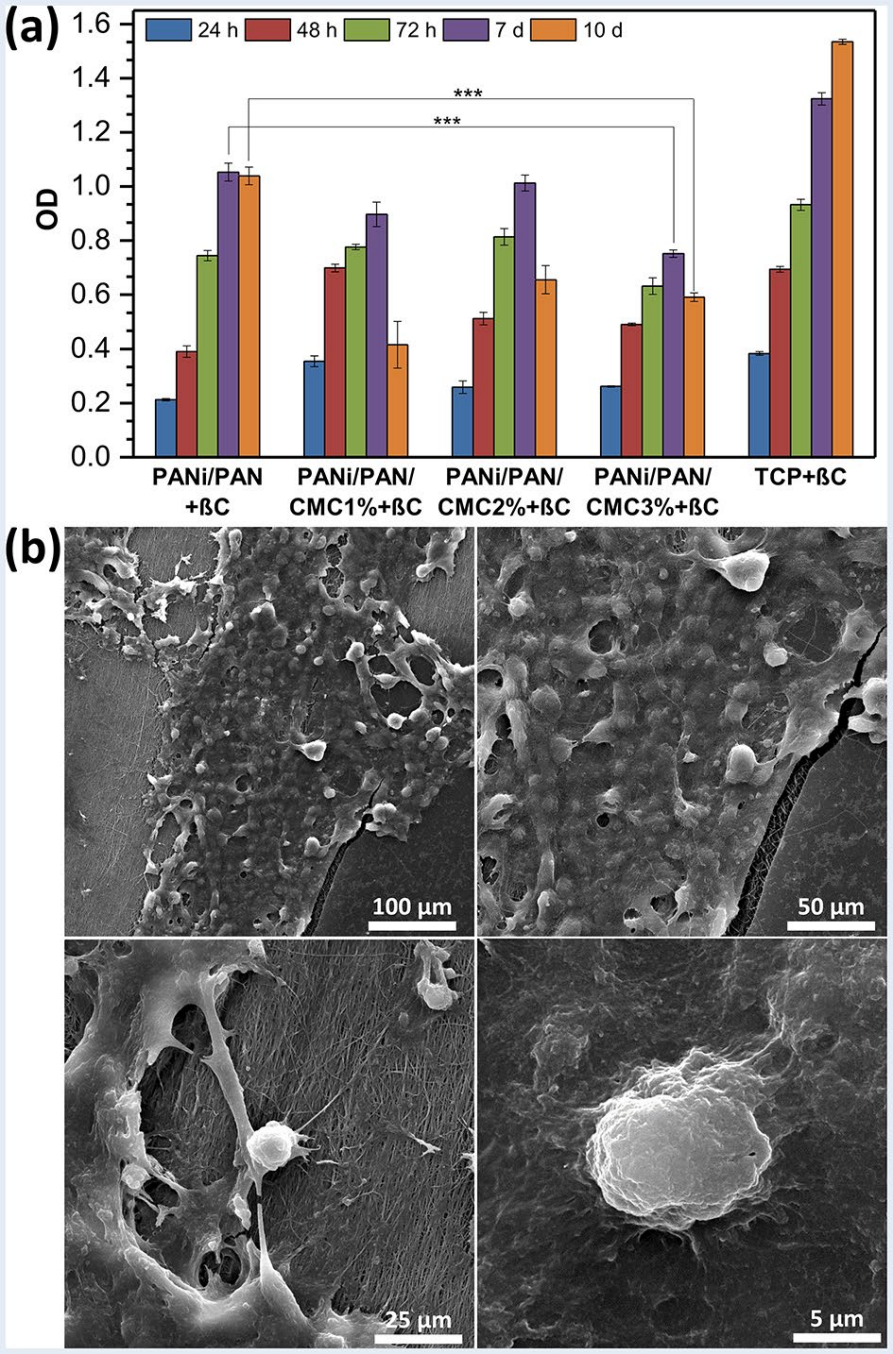
(**a**) The cell viability of hADMSCs (10^4^ cells/0.5 × 0.5 cm^2^ scaffold) cultured on scaffolds up to 10 d in the presence of βC (20 µM) obtained from MTT assay. ***: *p* < 0.001. (**b**) SEM images of hADMSCs cultured on scaffold PANi/PAN/CMC3%+βC for 10 d

The effects of βC as an efficient natural neurogenic inducer in combination with CMC-functionalized electroconductive PANi/PAN-based scaffolds on the neural differentiation hADMSCs were investigated. The MTT results (Fig. 4a) confirmed the viability and metabolic activity of the differentiated hADMSCs on the scaffolds cultured in the nutrient medium containing βC (20 µM), denoted as scaffolds PANi/PAN + βC, PANi/PAN/CMC1%+βC, PANi/PAN/CMC2%+βC, and PANi/PAN/CMC3%+βC. Up to 7 d of incubation, continuous prefiltration of cells on all scaffolds was observed. After 10 d of incubation, the cell viability for cells cultured on scaffolds PANi/PAN/CMC1%+βC, PANi/PAN/CMC2%+βC, and PANi/PAN/CMC3%+βC was significantly decreased (*p* < 0.001), while the cells on scaffold PANi/PAN + βC and TCP continued to proliferate. The decrease in cell viability for scaffolds PANi/PAN/CMC1%+βC, PANi/PAN/CMC2%+βC, and PANi/PAN/CMC3%+βC, amplified at higher surface-immobilized CMC contents, was not as a result of a cytotoxicity effect, but due to the switch from proliferation to differentiation [20, 22, 25–27]. SEM images (Fig. 4b) showed that the morphology of hADMSCs cultured on scaffold PANi/PAN/CMC3%+βC for 10 d was transformed to neuron-like cells, while the cells positioned between nanofibers and exposed good cell-cell and cell-scaffold interactions.

**Fig. 5:**
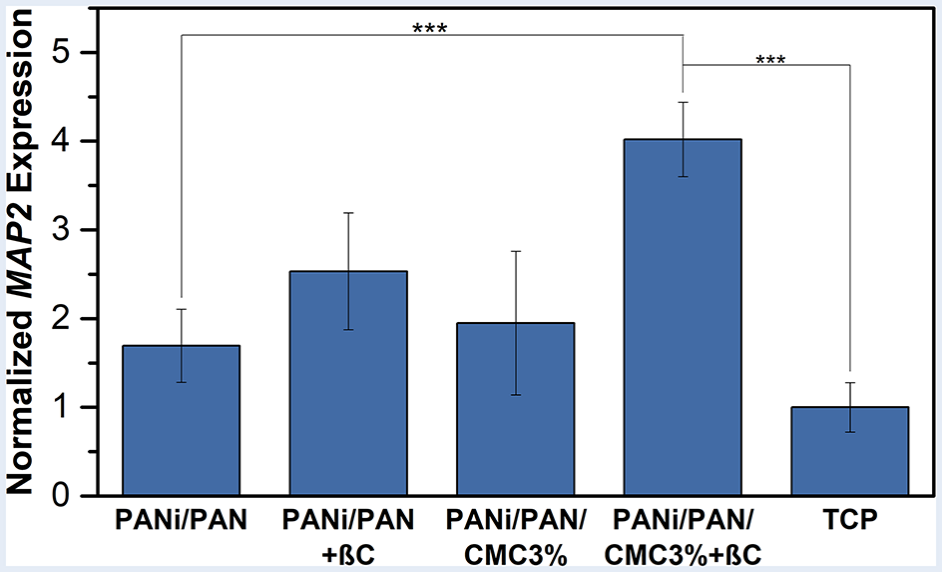
Normalized expression of *MAP2* gene in hADMSCs (10^4^ cells/0.5 × 0.5 cm^2^ scaffold) cultured on scaffolds for 10 d in the presence or absence of βC (20 µM) obtained from qRT-PCR. ***: *p* < 0.001

The neurogenic induction for the differentiated cells was quantified by assessing the expression of *MAP2* genes in mRNA levels through qRT-PCR and normalizing with the expressions of the *GAPDH* gene as a control gene (Fig. 5). *MAP2* gene is one of the most significant in vitro markers for mature neurons, investigated in the course of the neurogenesis process study [33]. The employment of βC and CMC-functionalization improved the level of *MAP2* expression for the differentiated cells on the scaffolds PANi/PAN + βC and PANi/PAN/CMC3%, respectively, compared to the scaffold PANi/PAN and TCP. Meanwhile, the combination of PANi-incorporation, CMC-functionalization, and βC displayed a synergic effect on the neurogenic induction, where a significantly higher level of *MAP2* expression (*p* < 0.001) was recorded for the differentiated cells on the scaffold PANi/PAN/CMC3%+βC comparing to others.

**Fig. 6:**
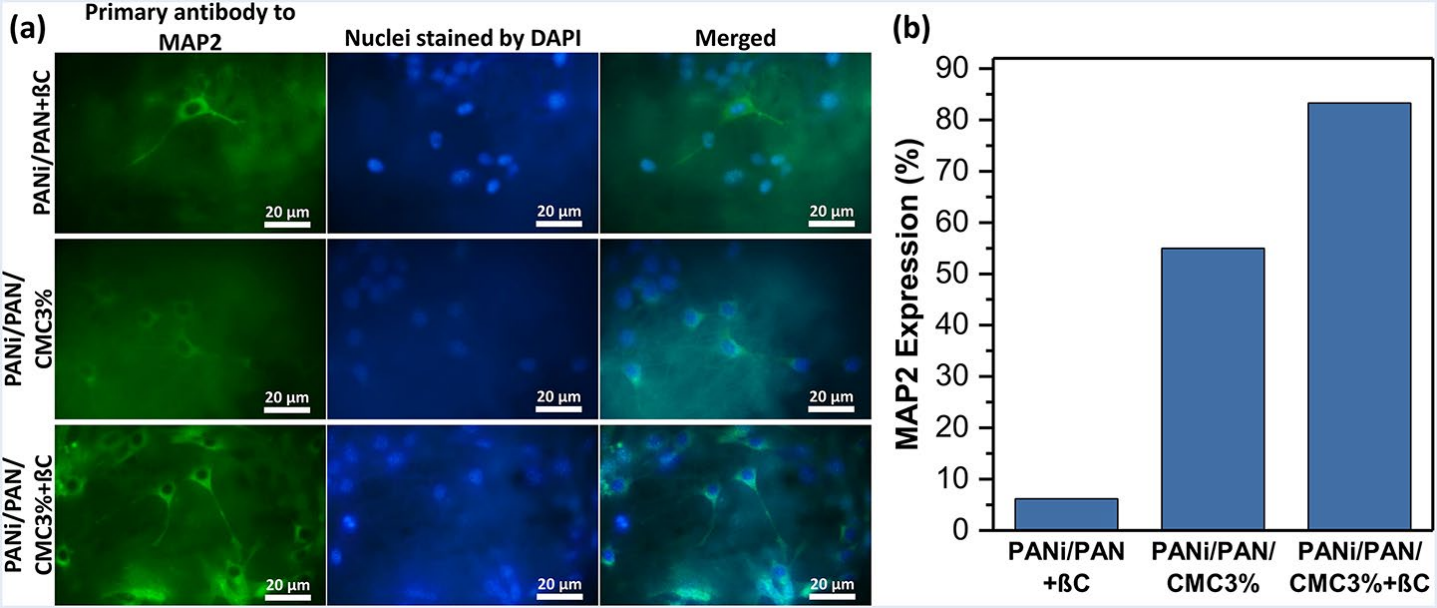
(**a**) Expression of MAP2 protein in hADMSCs (10^4^ cells/0.5 × 0.5 cm^2^ scaffold) cultured on scaffolds for 10 d in the presence or absence of βC (20 µM) obtained from ICC assay. (**b**) Quantification of fluorescence microscopy images

Finally, the expression of MAP2 at the protein level for the differentiated cells was studied by ICC assay. MAP2 is a neuronal phosphoprotein that regulates the structure and stability of microtubules, neuronal morphogenesis, cytoskeleton dynamics, and organelle trafficking in axons and dendrites. Isoforms of MAP2 are expressed in the perikarya and dendrites of neurons. The fluorescence microscopy images (Fig. 6a) demonstrated the expression of MAP2 protein for scaffolds PANi/PAN + βC, PANi/PAN/CMC3%, and PANi/PAN/CMC3%+βC. The presence of βC and CMC provided a neuro-inductive environment for the differentiation of hADMSCs into neuron-like cells. Meanwhile, the cells resembled nerve cells in morphology. DAPI staining showed the healthiness of cells’ nuclei. Similar to qRT-PCR results, the combination of βC and CMC-functionalization resulted in a synergic neurogenic induction effect, where a significantly higher level of the expression for MAP2 protein (*p* < 0.001) was detected for the differentiated cells on the scaffold PANi/PAN/CMC3%+βC (83%) comparing to others (Fig. 6b).

## Conclusion

The surface of PANi/PAN nanofibers can be functionalized with CMC to fabricate scaffolds for nerve tissue engineering applications. The FTIR, SEM, and water contact angle results showed the successful functionalization process. The microscopy and MTT data confirmed the biocompatible of the fabricated scaffolds to support the attachment, proliferation, and differentiation of seeded hADMSCs. The CMC-functionalized PANi/PAN-based scaffolds could provide a suitable microenvironment to induce neurogenesis for hADMSCs in the presence of βC as a natural neural differentiation agent. The qRT-PCR and ICC results exhibited the synergic neurogenic induction effect of CMC-functionalization and βC. The neuro-inductivity of the surface-immobilized CMC on the scaffolds was concentration-dependent.

As a research limitation, witnessing the complete neurogenesis of hADMSCs into mature neurons, oligodendrocytes, or astrocytes was not possible in this in vitro study; although the expression of MAP2 (a marker of mature neurons) confirmed the neurogenic induction on the cells cultured on the scaffold PANi/PAN/CMC3%+βC for 10 d. The neural differentiation could be more quantitated by investigating the expression of other genes such as *Myelin basic protein* (*MBP*, a marker of oligodendrocytes) and *Glial fibrillary acidic protein* (*GFAP*, a marker of neural progenitor cells, NPCs, and astrocytes). This study can be further extended by an in vivo study, by exploring the mechanisms of the neurogenesis process induced via surface-immobilized CMC moieties or by comparing the results with a generally used substrate such as fibronectin/Poly-L-Lysine as a positive control.

## Data Availability

All data generated or analyzed during this study are included in the manuscript.

## References

[1] Guo B, Ma PX (2018). Conducting polymers for tissue Engineering. Biomacromolecules.

[2] Orafa Z, Bakhshi H, Arab-Ahmadi S, Irani S (2022). Laponite/amoxicillin-functionalized PLA nanofibrous as osteoinductive and antibacterial scaffolds. Scientific Reports.

[3] Orafa Z, Irani S, Zamanian A, Bakhshi H, Nikukar H, Ghalandari B (2021). Coating of Laponite on PLA Nanofibrous for bone tissue Engineering Application. Macromolecular Research.

[4] Jadoun, S., Riaz, U., & Budhiraja, V. (2021). Biodegradable conducting polymeric materials for biomedical applications: A review. *MEDICAL DEVICES & SENSORS*, *4*(1), 10.1002/mds3.10141.

[5] Jiang L, Chen D, Wang Z, Zhang Z, Xia Y, Xue H, Liu Y (2019). Preparation of an electrically conductive graphene Oxide/Chitosan Scaffold for Cardiac tissue Engineering. Applied Biochemistry and Biotechnology.

[6] Deliormanlı AM, Atmaca H (2018). Biological Response of Osteoblastic and Chondrogenic cells to Graphene-Containing PCL/Bioactive Glass Bilayered Scaffolds for Osteochondral tissue Engineering Applications. Applied Biochemistry and Biotechnology.

[7] Kheilnezhad B, Firoozabady S, Aidun A (2020). An overview of polyaniline in tissue Engineering. Journal of Tissues and Materials.

[8] Hosseinzadeh S, Mahmoudifard M, Mohamadyar-Toupkanlou F, Dodel M, Hajarizadeh A, Adabi M, Soleimani M (2016). The nanofibrous PAN-PANi scaffold as an efficient substrate for skeletal muscle differentiation using satellite cells. Bioprocess and Biosystems Engineering.

[9] Wang M, Tremblay PL, Zhang T (2021). Optimizing the electrical conductivity of polyacrylonitrile/polyaniline with nickel nanoparticles for the enhanced electrostimulation of Schwann cells proliferation. Bioelectrochemistry.

[10] Mohamadali M, Irani S, Soleimani M, Hosseinzadeh S (2017). PANi/PAN copolymer as scaffolds for the muscle cell-like differentiation of mesenchymal stem cells. Polymers for Advanced Technologies.

[11] Toptaş N, Karakışla M, Saçak M (2009). Conductive polyaniline/polyacrylonitrile composite fibers: Effect of synthesis parameters on polyaniline content and electrical surface resistivity. Polymer Composites.

[12] Zhang X, Li L, Ouyang J, Zhang L, Xue J, Zhang H, Tao W (2021). Electroactive electrospun nanofibers for tissue engineering. Nano Today.

[13] Rahmati M, Mills DK, Urbanska AM, Saeb MR, Venugopal JR, Ramakrishna S, Mozafari M (2021). Electrospinning for tissue engineering applications. Progress in Materials Science.

[14] Zhang X, Meng Y, Gong B, Wang T, Lu Y, Zhang L, Xue J (2022). Electrospun nanofibers for manipulating soft tissue regeneration. Journal of Materials Chemistry B.

[15] Seidi F, Khodadadi Yazdi M, Jouyandeh M, Dominic M, Naeim H, Nezhad MN, Mozafari M (2021). Chitosan-based blends for biomedical applications. International Journal of Biological Macromolecules.

[16] Rajabi M, McConnell M, Cabral J, Ali MA (2021). Chitosan hydrogels in 3D printing for biomedical applications. Carbohydrate Polymers.

[17] Arab-Ahmadi S, Irani S, Bakhshi H, Atyabi F, Ghalandari B (2021). Immobilization of carboxymethyl chitosan/laponite on polycaprolactone nanofibers as osteoinductive bone scaffolds. Polymers for Advanced Technologies.

[18] Arab-Ahmadi S, Irani S, Bakhshi H, Atyabi F, Ghalandari B (2021). Immobilization of cobalt‐loaded laponite/carboxymethyl chitosan on polycaprolactone nanofiber for improving osteogenesis and angiogenesis activities. Polymers for Advanced Technologies.

[19] Alemi PS, Atyabi SA, Sharifi F, Mohamadali M, Irani S, Bakhshi H, Atyabi SM (2019). Synergistic effect of pressure cold atmospheric plasma and carboxymethyl chitosan to mesenchymal stem cell differentiation on PCL/CMC nanofibers for cartilage tissue engineering. Polymers for Advanced Technologies.

[20] Shapourzadeh A, Atyabi SM, Irani S, Bakhshi H (2020). Osteoinductivity of polycaprolactone nanofibers grafted functionalized with carboxymethyl chitosan: Synergic effect of β-carotene and electromagnetic field. International Journal of Biological Macromolecules.

[21] Kabirkoohian A, Bakhshi H, Irani S, Sharifi F (2022). Chemical immobilization of Carboxymethyl Chitosan on Polycaprolactone Nanofibers as Osteochondral Scaffolds. Applied Biochemistry and Biotechnology.

[22] Sharifi F, Atyabi SM, Irani S, Bakhshi H (2020). Bone morphogenic protein-2 immobilization by cold atmospheric plasma to enhance the osteoinductivity of carboxymethyl chitosan-based nanofibers. Carbohydrate Polymers.

[23] Sharifi F, Atyabi SM, Norouzian D, Zandi M, Irani S, Bakhshi H (2018). Polycaprolactone/carboxymethyl chitosan nanofibrous scaffolds for bone tissue engineering application. International Journal of Biological Macromolecules.

[24] Mohammadnezhad J, Khodabakhshi-Soreshjani F, Bakhshi H (2016). Preparation and evaluation of chitosan-coated eggshell particles as copper(II) biosorbent. Desalination and Water Treatment.

[25] Dabouian A, Bakhshi H, Irani S, Pezeshki-Modaress M (2018). β-Carotene: A natural osteogen to fabricate osteoinductive electrospun scaffolds. RSC Advances.

[26] Esmailian S, Irani S, Bakhshi H, Zandi M (2018). Biodegradable bead-on-spring nanofibers releasing β-carotene for bone tissue engineering. Materials Science and Engineering: C.

[27] Moradi Y, Atyabi SA, Ghiassadin A, Bakhshi H, Irani S, Atyabi SM, Dadgar N (2022). Cold Atmosphere plasma modification on Beta-carotene-loaded Nanofibers to enhance osteogenic differentiation. Fibers and Polymers.

[28] Masek A, Chrzescijanska E, Marian Zaborski (2015). Application of β-carotene, a natural flavonoid dye, to polymeric materials as a natural antioxidant and determination of its characteristics using cyclic voltammetry and FTIR spectroscopy. International Journal of Electrochemical Science.

[29] Leatherman G, Durantini EN, Gust D, Moore TA, Moore AL, Stone S, Lindsay SM (1999). Carotene as a Molecular Wire: Conducting Atomic Force Microscopy. The Journal of Physical Chemistry B.

[30] Asghari N, Irani S, Pezeshki-Moddaress M, Zandi M, Mohamadali M (2022). Neuronal differentiation of mesenchymal stem cells by polyvinyl alcohol/Gelatin/crocin and beta-carotene. Molecular Biology Reports.

[31] Magalingam KB, Somanath SD, Haleagrahara N, Selvaduray KR, Radhakrishnan AK (2022). Unravelling the neuroprotective mechanisms of carotenes in differentiated human neural cells: Biochemical and proteomic approaches. Food Chemistry: Molecular Sciences.

[32] Dadashkhan S, Irani S, Bonakdar S, Ghalandari B (2021). P75 and S100 gene expression induced by cell-imprinted substrate and beta‐carotene to nerve tissue engineering. Journal of Applied Polymer Science.

[33] Hazeri Y, Irani S, Zandi M, Pezeshki-Modaress M (2020). Polyvinyl alcohol/sulfated alginate nanofibers induced the neuronal differentiation of human bone marrow stem cells. International Journal of Biological Macromolecules.

[34] Lee HA, Park S, Kim Y (2013). Effect of β-carotene on cancer cell stemness and differentiation in SK-N-BE(2)C neuroblastoma cells. Oncology Reports.

[35] Holland ER, Pomfret SJ, Adams PN, Monkman AP (1996). Conductivity studies of polyaniline doped with CSA. Journal of Physics: Condensed Matter.

[36] Eren O, Ucar N, Onen A, Kizildag N, Karacan I (2016). Synergistic effect of polyaniline, nanosilver, and carbon nanotube mixtures on the structure and properties of polyacrylonitrile composite nanofiber. Journal of Composite Materials.

[37] Prabhakaran MP, Ghasemi-Mobarakeh L, Jin G, Ramakrishna S (2011). Electrospun conducting polymer nanofibers and electrical stimulation of nerve stem cells. Journal of Bioscience and Bioengineering.

[38] Jun I, Jeong S, Shin H (2009). The stimulation of myoblast differentiation by electrically conductive sub-micron fibers. Biomaterials.

[39] Jeong SI, Jun ID, Choi MJ, Nho YC, Lee YM, Shin H (2008). Development of Electroactive and Elastic Nanofibers that contain polyaniline and poly(L-lactide-co-ε-caprolactone) for the control of cell adhesion. Macromolecular Bioscience.

[40] Duester G (2000). Families of retinoid dehydrogenases regulating vitamin A function: Production of visual pigment and retinoic acid. European Journal of Biochemistry.

[41] Duester G (2008). Retinoic acid synthesis and signaling during early Organogenesis. Cell.

